# Open stented elephant trunk for complicated Stanford type B aortic dissection: a single-center experience

**DOI:** 10.1186/s13019-020-01341-6

**Published:** 2020-09-29

**Authors:** Hongtao Tie, Lingwen Kong, Zhengjie Tu, Dan Chen, Delai Zheng, Qingchen Wu, Qiang Li

**Affiliations:** 1 Department of Cardiothoracic Surgery, The First Affiliated Hospital of Chongqing Medical University, Chongqing, 400016 China; 2 Department of Cardiothoracic Surgery, Chongqing Medical Emergency Center, Chongqing, 400014 China

**Keywords:** Complicated Stanford type B aortic dissection, Stented elephant trunk, Subclavian artery correction

## Abstract

**Background:**

Open stented elephant trunk (SET) or SET with left subclavian artery (LSCA) to left common carotid artery (LCCA) bypass is proven to a potentially alternative treatment for complicated Stanford type B aortic dissection (TBAD). In the current study, we reported our experience with ten consecutive TBAD patients who underwent open SET.

**Methods:**

Patients with complicated TBAD underwent open SET from May 2016 to November 2018 in our institution were included. Patients’ clinical data were obtained from the electronic medical record system, and long-term clinical outcomes were collected by telephone interviews or outpatient interviews.

**Results:**

A total of ten patients with nine males and one female were included, and the average age was 47.3 (31–65) years. Increased D-dimer and fibrinogen degradation products were observed in all patients at admission, and two patients had renal insufficiency. The average postoperative mechanical ventilation time, length of stay in intensive care unit, and postoperative hospital length of stay were 46.9 (6.7–151.2) hours, 7.7 (4–17) days, and 15.7 (10–26) days. No postoperative death occurred. Acute kidney injury and other complications were observed, and they were recovered well when discharge. In long-term follow-up, computed tomography angiography indicated that aortas were completely well remodeled, and blood supply of the brachiocephalic trunks was normal without anastomotic complications. All patients lived well.

**Conclusion:**

SET or SET with subclavian artery correction shows satisfactory clinical outcomes, and it could be considered as an alternative treatment. Well-designed, large-scale studies with long-term follow-up are still needed.

## Background

Aortic dissection (AD) is a life-threatening disease, characterized by rapid blood flowing into the media and separating the intima and the adventitia apart. It was estimated that the annual incidence of AD ranged from 2·9 to 3·5 per 100,000 population [1]. The actual incidence is higher because of deaths before hospital admission [2, 3] and increasing with the population aging [4]. About 25 to 40% AD is type Stanford type B, characterized by an intimal tear in the descending aorta without the extension of ascending aorta involved. Stanford type B AD (TBAD) is classified as uncomplicated and complicated ones. Though TBAD is tended to have a stable in-hospital course, complicated TBAD is associated with fatal complications and early mortality.

Complicated TBAD is defined by the presence of persistent or recurrent pain, uncontrolled hypertension despite medication treatment, aortic expansion, organ malperfusion, signs of a rupture, location of the intimal tear or retrograde dissection into aortic arch [5, 6]. The best medical treatment, open surgery, and endovascular treatment are three routine ways to treatment TBAD, while both thoracic endovascular aortic repair (TEVAR) and open surgery are recommended for complicated TBAD [7]. In recent decades, TEVAR has become the mainstream for treatment of complicated TBAD owing to its favorable short and mid-term outcomes. TEVAR is recommended for patients with complicated TBAD by the European Society of Cardiology guidelines [8]. Nevertheless, complicated TBAD patient with visceral malperfusion still has a poorer prognosis [9]. And TEVAR is also associated with unfavorable consequences, such as aortic injury, retrograde ascending aortic dissection, new-onset intimal tear during the procedure, endoleak, and stent graft infoldings, collapse or migration in long-term follow-up [10–12]. Additionally, for cases with abnormal femoral and iliac arteries, connective tissue diseases, and intimal tear near to or dissection extension to the origin of the left subclavian artery (LSCA), open surgery becomes an appropriate treatment.

Previous studies have proven satisfactory clinical results of open stented elephant trunk (SET) implantation and open SET with LSCA to the left common carotid artery (LSCA-LCCA) bypass [13–15] for complicated TBAD patients. However, because of rare cases used this treatment, the surgical method still needs to be evaluated. In the current study, we reported our experience with ten consecutive patients who underwent open SET for complicated TBAD patients.

## Methods

### Patients

This retrospective cohort study was approved by the Institutional Review Board of our institution, and we reported the study in according to the STROCSS criteria [16]. Patients were diagnosed as complicated TBAD indicated by the aorta computed tomography angiography (CTA) and echocardiography. And patients received open SET with or without subclavian artery between May 2016 and November 2018 in our hospital were included. Data of patients’ baseline demographics and inspection results before surgery were obtained from the electronic medical record system.

### Surgical procedure

After tracheal intubation and anesthesia, left radial artery catheterization, central vein catheterization, and left dorsalis pedis artery catheterization were performed to monitor. Innominate artery, LCCA, LSCA, and the transverse arch were freed from surrounding tissue by a median sternotomy incision. Right axillary artery and venous cannulation of the right atrium were used for cardiopulmonary bypass (CPB) support, and selective cerebral perfusion was achieved by the right axillary artery. The aorta was clamped when nasopharyngeal temperature reached 30–33 °C and cardioplegic solution was imported into the coronary artery for cardiac arrest. Brachiocephalic vessels were clamped and circulatory arrest was achieved when nasopharyngeal temperature reached 22–26 °C, and brain protection was performed by antegrade selective cerebral perfusion via right axillary artery at approximately 5 to 10 ml/kg.min. Then a half circumferential incision was done in the anterior wall of the aortic arch, and a self-expandable stented graft (Microport, Shanghai, China) was implanted near LSCA via the incision to seal the intimal tear thoroughly. After that, the proximal stented graft was circumferentially sutured to be fixed on the normal aortic arch wall with 4–0 Prolene, and the aortic arch incision was also continuously sutured with 4–0 Prolene. Rewarming began, and CPB support was gradually returned to normal flow.

**Fig. 1:**
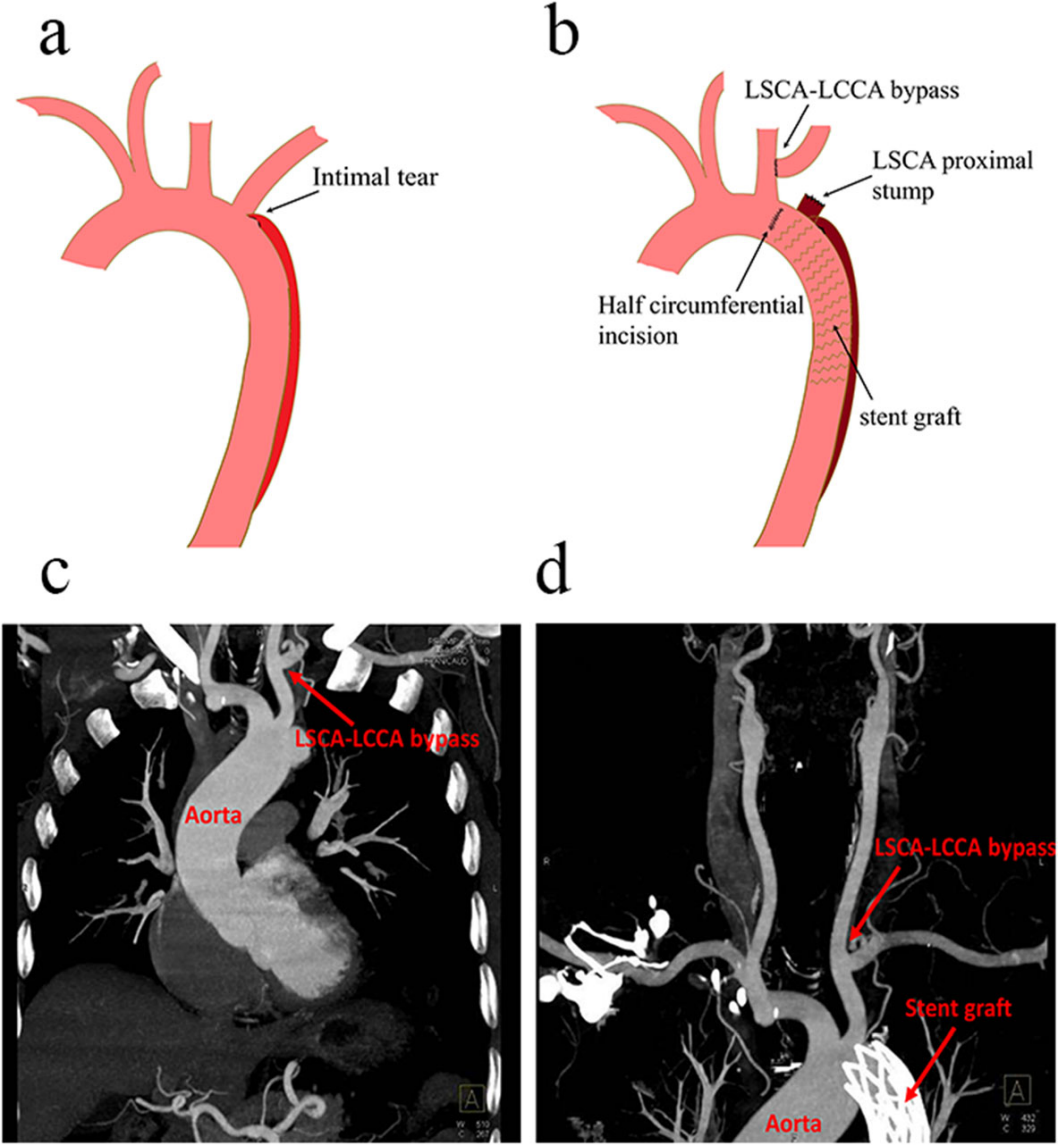
Schematic illustration of the surgery and patients’ postoperative CT. **a** Intimal tear near to the origin of the left subclavian artery; **b** Schematic illustration of SET with LSCA-LCCA bypass; **c**, **d** postoperative CT of patietns after surgery

If patients accompanied with dissection extension to LSCA, intimal tear near to LSCA, (Fig. 1a) or aberrant right subclavian artery (RSCA), LSCA bypass or aberrant RSCA correction would be operated. After free from the surrounding tissue, proximal stump of LSCA or aberrant RSCA was sutured and the distal stump was anastomosed to the common carotid artery with an end-to-side method. After returning to normal temperature, CPB was stopped, cannulas were gradually removed, and median sternotomy was routinely closed. A schematic illustration of the surgery procedure is described in Fig. 1b.

### Postoperative care and follow-up

Patients were delivered to the intensive care unit (ICU) with routine monitoring and interventions after surgery. Perioperative data, postoperative data, complication data, and long-term clinical outcomes were collected from the medical record and through telephone or outpatient interviews to patients or family members. After discharge, patients returned to the hospital for routine assessment at postoperative 3 months, 6 months, 12 months, and subsequently annually. The routine inspection assessments involved aorta CTA, echocardiography, and electrocardiogram.

### Statistical analysis

Data was presented as mean and range for continuous variable and count with percentage for categorical variable. All statistical analyses were performed using software SPSS Statistics 21.0 (IBM Corporation, Armonk, NY, USA).

## Results

### Baseline characteristics

A total of ten patients with complicated TBAD were enrolled, including nine males and one female. Among them, eight were acute and two were chronic TBAD. The average age was 47.3 years (31–65). Table 1 shows the detailed characteristics of the ten patients. Preoperative routine chemical examination revealed normal accounts of platelet, increased D 2dimer and increased fibrinogen degradation products in all patients, and renal insufficiency in two patients. Preoperative imagological examination indicated that mild ascending aorta dilatation, aberrant RSCA, dissection involving or intima tear near the LSCA, dissection involving the renal artery, left ventricular hypertrophy, and dissection aneurysm with thrombus were accompanied.

**Table 1:** Patients baseline characteristics

Variables	n(%)/Mean(R)
Gender (Male)	9 (90%)
Age (years)	47.3 (31–65)
Height (cm)	166.6 (140–177)
Weight (kg)	73 (46–90)
BMI (Kg/m2)	26.1 (21.3–31.1)
Heart beat at admission (bpm)	89.8 (63–105)
Systolic blood pressure at admission (mmHg)	161.2 (117–224)
Diastolic blood pressure at admission (mmHg)	97.7 (77–142)
Hypertension	10 (100%)
diabetes mellitus	0
Coronary artery disease	1 (10%)
current smoker	8 (80%)
Drink	6 (60%)
Platelet count at admission (X10^9)	244.1 (94–403)
D dimer (mg/L FEU)	3.8 (1.5–13.1)
FDP (ug/ml)	11.3 (3.8–33.5)
Serum creatinine (umol/L)	92.9 (47–208)
Mild ascending aorta dilatation	2 (20%)
Right subclavian artery vagus	2 (20%)
Lesions involving left subclavian artery	4 (40%)
Lesions involving renal artery	3 (30%)
Left ventricular hypertrophy,	7 (70%)
Dissection aneurysm with thrombus	1 (10%)

*BMI* Body Mass Index, *FDP* fibrinogen degradation product

### Intraoperative variables

All the ten patients underwent surgery by using moderate hypothermic circulatory arrest and antegrade cerebral perfusion. Six patients received SET with LSCA-LCCA, two received SET with LSCA-LCCA bypass and aberrant RSCA correction (aberrant RSCA-RCCA bypass), and the other two only received SET. The details of CPB duration, mean aortic clamp time, mean cerebral perfusion time, and intraoperative transfusion are displayed in Table 2.

**Table 2:** Intraoperative variables

Variables	Mean (R)
CPB duration (mins)	139.1 (121–169)
Aortic cross clamp (mins)	68.7 (43–100)
Mean cerebral perfusion time (mins)	42.0 (32–55)
RBC transfusion (U)	340 (0–400)
Plasma transfusion (ml)	490 (0–800)
Platelet transfusion (U)	2.2 (0–3)
Cryoprecipitate transfusion (U)	1 (0–6)
Urine volume (ml)	575 (40–1000)

*CPB* cardiopulmonary bypass, *RBC* red blood cell

### Postoperative complications

All surgeries were performed successfully, and no death was observed. Myocardial injury, postoperative atrial fibrillation, postoperative ventricular fibrillation, acute kidney injury (AKI), postoperative liver dysfunction, re-intubation, mechanical ventilation (MV) time > 48 h, postoperative delirium was observed, as presented in Table 3. Among the two patients with AKI, one gradually recovered, the other one received continuous renal replacement therapy (CRRT) for 50 days and subsequently be treated by oral medication.

**Table 3:** Early postoperative complications

Variable	n (%)
Myocardial injury	1 (10%)
Atrial fibrillation	1 (10%)
Ventricular fibrillation	1 (10%)
Renal insufficiency	1 (10%)
Acute kidney injury	2 (20%)
Liver dysfunction	7 (70%)
CRRT	1 (10%)
Intratracheal intubation again	1 (10%)
Mechanical ventilation time > 48 h	3 (30%)
Delirium	1 (10%)

Renal insufficiency: serum creatinine >133umol/L; acute kidney injury: Scr>226 umol/L or CRRT; Liver dysfunction: elevated aspartate aminotransferase or alanine aminotransferase with elevated total bilirubin or direct bilirubin after surgery; Myocardial injury: cTnT> 0.2 μg/L. CRRT, continuous renal replacement therapy

The average postoperative MV time was 46.9 (6.7–151.2) hours, since three patients had relatively long MV times of 84.7 h, 95.8 h, and 151.2 h. The average length of stay in ICU was 7.7 (4–17) days, with 3 patients more than 9 days because of CRRT therapy, pneumonia and re-intubation, and postoperative delirium. The mean postoperative hospital length of stay was 15.7 (10–26) days, and the average total hospitalization cost was 268,909 RMB (142,888 to 527,642).

### Prognosis

Postoperative 3 months, CTA showed that intima tear entry was completely closed without endoleak and the blood supply of the brachiocephalic trunks was normal without anastomotic complications, as shown in Fig. 1c and d. All ten patients were retrospectively interviewed, and follow-ups ranged from 10 to 40 months after discharge. Postoperative complications are all recovered except one patient with AKI needs long-term oral medication treatment. They lived well with a relatively normal life, and aortas are completely well remodeled indicated by CTA.

## Discussion

Complicated TBAD is challenging because its formidable risk of malperfusion, AD progression, and aorta rupture. The in-hospital mortality of patients with complicated TBAD was reported to be nearly 50%, while 10% for uncomplicated TBAD [17, 18]. Many interventions have been used to improve the survival of complicated TBAD patients, with none becoming the predominant therapy. In the current study, our single-center experience of open SET with subclavian artery for treatment of complicated TBAD patients shows a satisfactory clinical outcome, indicating that it is an alternative reliable treatment.

In patients with uncomplicated TBAD, the disease course can be safely stabilized via controlling the pain, blood pressure, and heart rate by medicine therapy. Current data show that TEVAR could improve aortic remodeling and decease disease progression and aorta-related mortality, but TEVAR manifests no clinical benefit on overall survival compared with medicine treatment for patients with uncomplicated TBAD [19, 20]. Even so, obliterating the intimal tear with membrane-covered stent-graft is the main treatment in clinical practice and TEVAR is also recommended for uncomplicated TBAD with a B level of evidence (Class of recommendation, IIa). While for complicated TBAD, both TEVAR and surgery therapy are recommended with a same evidence level of C [8]. In a retrospective study and meta-analysis, the author found that TEVAR and open surgical repair showed a similar long-term survival [21]. A report from the international registry of acute aortic dissections indicated that TEVAR is associated with better short-term outcomes of in-hospital mortality and complications [22]. With the minimally invasive nature and better clinical outcomes, TEVAR has been the preferred procedure for complicated TBAD.

A majority of patients with complicated TBAD could be treated with satisfactory results. However, in clinical practice, the anatomic complexity of aorta and branches, dissection location, and aortic arch angle often limit the use of the TEVAR, and TEVAR alone could not control the dissection progression and even cause severe complications. A proximal landing zone with a least of 1.5 cm between the intimal tear or dissection and the origin of the LSCA is necessary for the safe and precise stent implantation. Without enough landing zone, stents shifting would lead to LSCA closure or endoleak, and need re-intervention [21]. Blocking LSCA to obliterate intimal tear completely was associated with increased incidence of stroke, upper limb ischemia, and endoleak [23–25]. TEVAR with additional assistive techniques, such as chimney technique and supra-arch branch vessel bypass, is a more proper therapeutic way for complicated TBAD. But it correlated with increased operation difficulty, radiation exposure of both doctor and patient, contrast dosage, and medical cost [26].

Sometimes open surgery repair is preferred because of anatomic contraindications, dissection extension without a proximal landing zone, and concomitant aortic lesion. Compared with TEVAR, open SET has advantages of accurate stent positioning and implanting, reduced risk of stent shift and endoleak, reduced intramural blood clots entering circulation via precise suture, and well aortic reconstruction through stent expanding induced aortic layers adhesion. In Sun and colleagues’ work, open surgery of total arch replacement with SET implantation showed favorable outcomes in both acute and chronic TBAD [13, 14]. Additionally, open SET technique for complicated TBAD exhibited a good outcome [27], indicating it as an alternative feasible and safe option. Another study reported that open surgery repair, and TEVAR has similar early complications and mortality, but open surgery repair has better long-term outcomes of fewer re-intervention and improved survival [21]. In our study, all ten patients received open SET technique with satisfactory clinical outcomes also supports that open SET is an alternative therapy for complicated TBAD.

For patients with TBAD and distal aortic arch involvement, Sun and colleagues reported a one-step technique of open SET with LSCA-LCCA bypass and achieved a satisfactory clinical outcome [15]. It has the advantages of avoiding graft-related complications via preserving the autologous normal aortic wall, completely closing the false lumen, easier and safer features than TEVAR, and avoiding proximal endoleaks and retrograde dissection by fixing stent graft firmly [15]. In this study, six patients received open SET with LSCA-LCCA bypass for complicated TBAD, which also confirmed the satisfactory clinical outcomes of this technique.

In our study, no in-hospital mortality occurred. Though 70% patients have postoperative liver dysfunction, they were all transient, and recover well when discharge. Postoperative MV time and ICU length of stay are much longer in our study by comparison with Zhu and colleagues’ work [15]. It is because that three patients in our study have complications of respiratory insufficiency, unstable circulation, pneumonia, AKI, re-intubation, and postoperative delirium. These complications might correlate with longer duration of CPB, aortic cross clamp time, selective cerebral perfusion in our study, and perioperation management, indicating that a good heart team necessitate both surgical skills and post-operation management. Two patients with AKI gradually recovered, with one completely recovery and one receiving long-term medication use. These outcomes of no mortality, acceptable complication, and good prognosis proved the efficacy of SET or SET with subclavian artery correction for patients with complicated TBAD. Limitations should be concerned in currents study. Only ten patients with different forms of TBAD were included; no control group was involved, and the follow-up is relatively short. Well-designed studies with control group, large sample, and long follow-up are warranted to further investigate the effect of SET with subclavian artery correction in complicated TBAD patients.

## Conclusion

SET or SET with subclavian artery correction shows satisfactory clinical outcomes with acceptable complications and good prognosis in patients with complicated TBAD, it could be considered as an alternative treatment. For limitations of small sample, no control group, and short follow-up, well-designed, large-scale studies with long-term follow-up are still needed.

## Data Availability

The datasets used and analyzed during the current study are available from the corresponding author on reasonable request.

## Abbreviations

AD: Aortic dissection
TBAD: Stanford type B AD
TEVAR: Thoracic endovascular aortic repair
SET: Stented elephant trunk
LSCA: Left subclavian artery
LCCA: Left common carotid artery bypass
RSCA: Right subclavian artery
CTA: Computed tomography angiography
CPB: Cardiopulmonary bypass
